# Travel Website Atmospheres Inducing Older Travelers’ Familiarity: The Moderating Role of Cognitive Age

**DOI:** 10.3390/ijerph18094812

**Published:** 2021-04-30

**Authors:** Soojung Kim, Yahua Bi, Insin Kim

**Affiliations:** 1 Institute of Economics and International Trade, Pusan National University, Busan 46241, Korea; ksoojung8014@gmail.com; 2 Department of Tourism and Convention, Pusan National University, Busan 46241, Korea; yahuabi@pusan.ac.kr

**Keywords:** older travelers, website atmosphere, familiarity, revisit intention, cognitive age

## Abstract

In the environment in which an increasing number of older travelers are participating in online tourism platforms, for older travelers who face multiple barriers in using e-commerce, it is essential to identify factors promoting older travelers’ website usage for their well-being and sustainable travel industry. This study aims to identify the key factors of website atmosphere for enhancing older travelers’ familiarity, investigate the relationship between older adults’ website familiarity and revisit intention, and test the moderating role of cognitive age. A web-based survey was conducted, and the sample consisted of 305 US residents 50 years of age and older who had experienced travel websites. The results indicated that three subdimensions of older travelers’ website familiarity—informativeness, effectiveness, and entertainment—positively influence their website familiarity. Additionally, the impact of informativeness on website familiarity is stronger for travelers who identify as younger than their chronological age. Moreover, older travelers’ familiarity with a website improves their revisit intention. The current study found not only significant travel website atmosphere factors to boost older travelers’ participation in online tourism platforms but also significant older travelers’ groups depending on their cognitive age perception to magnify the effect of website atmosphere.

## 1. Introduction

The number of older travelers continues to grow worldwide, with baby boomers who were born between 1946 and 1964 emerging as a vital travel market [1,2]. Older travelers have been identified as one of the most profitable travel markets [3,4,5] because today’s seniors are healthier, richer, more educated, and more independent than those in previous generations, which results in them placing a higher priority on travel in their retirement years [6,7]. Moreover, older travelers experience positive emotions, which potentially foster well-being outcomes and increase spirituality, which is a central component of well-being at old age [8,9], resulting in older travelers becoming a growing segment in the travel market.

The increasing availability of information and communication technologies (ICTs) has affected tourism and travel industries, and in particular, the way tourists explore, plan, and purchase tourism products [10]. Instead of visiting a travel agent, tourists now visit tourism websites to obtain travel information, plan their travel, and purchase travel products by themselves [11]. Given the increase in the advent of travel websites, some studies have highlighted the importance of website atmosphere on online experiences. For example, Björk [12] found that elements of website atmosphere such as information and pictures are effective in creating positive emotions such as being excited, charmed, and/or satisfied. Gao and Bai [13] argued that website atmosphere impacts the development of flow, which subsequently results in consumers’ satisfaction and purchase intention. Although there has been some research into website atmosphere, older adults are still one of the neglected segments even though older travelers are turning to internet users to explore and purchase tourism products [14,15,16,17].

An increasing portion of older adults participate in travel; however, the percentage of ICT users among older people is still much lower than among younger age groups [18]. Older adults are less familiar with the internet and experience computer anxiety and uncertainty during internet usage, which discourages them from active internet usage [19,20,21]. To reduce the uncertainty and challenges associated with using the internet, Marquié et al. [22] emphasized website familiarity for the older adults. Website familiarity increases the users’ confidence in browsing and purchasing online product [19] and affects consumers’ experiences and evaluations of websites [23] such as building online trust [24]. Since the importance of website familiarity has been emphasized in fostering older adults’ active internet usage, this study explores the components of website atmospherics that have a significant impact on building familiarity for older travelers using travel websites.

More importantly, although older adults were found to show different behaviors and attitudes toward their lifestyle, the studies exploring ICT use in older adults have mostly adopted chronological age [18]. Older adults tend to have different cognitive and chronological ages [25], and their use of innovative services (i.e., online websites) varies depending on their cognitive age [26]. Eastman and Iyer [27] argued that cognitive age may be a better measure for understanding and segmenting the older market. Older consumers show distinctive perceptions and behaviors in e-commerce that vary by cognitive age, which can amplify the effect of website atmosphere on familiarity. Therefore, this study adopts the concept of cognitive age in the exploration of older travelers’ e-commerce usage. 

To deepen our understanding of how website atmosphere creates familiarity based on older travelers’ perspectives, this study examines the impact of the three atmosphere elements (i.e., informativeness, effectiveness, and entertainment) on older travelers’ familiarity and their intention to revisit travel websites. This study also investigates the moderating role of cognitive age on the relationship between website atmosphere and older travelers’ familiarity. This exploration of older travelers will contribute to the design and construction of online travel websites for older travelers, which is an emerging profitable tourism market. Moreover, adopting the concept of cognitive age will enable researchers to segment the older travelers’ market in a more specific and practical way.

## 2. Literature Review

### 2.1. Familiarity

Consumers’ familiarity plays a crucial role in positive online consumer behavior by building and strengthening long-term relationships that lead users to regularly visit and remain loyal to a website [23,28,29,30,31]. Familiarity can be understood with both cognitive [32] and emotional approaches [33,34]. From the cognitive perspective, website familiarity is understood with the perceived ease of, knowledge, and skills about the information process practice in the website. From the emotional and affective perspectives, website familiarity refers to a user’s feeling of closeness with and understanding of web-based services [28]. 

Cho et al. [35] reported that familiarity is associated with perceived controllability and prior experience. Perceived controllability refers to a user’s judgment of their ability to obtain positive outcomes and avoid negative outcomes through their actions [36]. For example, older travelers have familiarity with a website when they can obtain positive outcomes such as gaining proper information and/or hedonic experiences. Prior experiences include positive or negative feedback based on personal experiences with the resource [37]. Positive prior experience increases individuals’ affinity for as well as their skills in using the website [38].

Moreover, evidence suggests that familiarity reduces uncertainty and builds trust in a website [39]. A lack of trust in a vendor and/or a website may result from consumers’ concerns regarding the quality of the product and/or payment security issues, which discourage consumers from engaging in online transactions [40,41]. Jo Black et al. [42] emphasized the importance of familiarity in risk reduction and found that a lack of familiarity leads to higher perceptions of risk with the internet than with other media. Gefen and Straub [39] argued that online consumer familiarity facilitates building users’ trust online that induces parasocial interaction [43]. Familiarity supports consumers by assuring them of their expected outcomes and that vendors care about consumers and will behave reliably by delivering goods and services on time and abiding by expected rules of conduct.

### 2.2. Online Website Atmosphere

Website atmosphere is a core part of tourism and hospitality marketing since it determines electronic service quality, and more importantly, it leads to consumers’ online purchases [13,44]. Baker et al. [45] emphasized the importance of website atmosphere in marketing by arguing that the website environment and atmosphere are more influential than other marketing activities at the point of purchase. In his seminal research, Kotler [46] defined atmosphere as the “conscious designing of the space to create specific effects on buyers” (p. 50). Atmosphere in tourism and hospitality marketing can be understood as stimuli that provide environmental signals affecting consumers’ behavior such as their actual and/or intention to purchase [13]. In an online context, web atmosphere refers to the “conscious designing of web environments to create positive effects in users in order to increase favorable consumer responses” [47].

Online website atmosphere can be manipulated to maximize positive outcomes. Kurniawan and Zaphiris [48] emphasized the need to build a website atmosphere that is suitable for older adults. The major factors related to website atmosphere are informativeness, effectiveness, and entertainment [13,49]. Informativeness and effectiveness are related to a cognitive approach and directly support the consumers’ achievement of a shopping goal, while entertainment is relatively inconsequential to the completion of a shopping goal [47]. 

#### 2.2.1. Informativeness

Consumers who purchase travel products online are mainly motivated by rich and varied information [50]. Informativeness refers to the amount and richness of the information contained in a website and includes information about products and/or transaction details such as payment options and shipping information [51]. Informativeness is the best predictor of positive attitudes [52,53], such as consumers’ perceived website quality and high satisfaction with the website [53].

Older adults generally tend to be risk averse and uncomfortable with uncertainty [54]; they are cautious and prefer to gather as much information as possible before making a decision [55]. Moreover, one of the major motivations in adopting new technology is obtaining new information related to one’s interests [21]. Thus, older adults are expected to feel an affinity for an informative website that provides useful, resourceful, and expert product information [13,51,53]. Moreover, older adults are interested in adopting technology when they perceive it to be useful [52]. Schlosser [56] argued that users develop a favorable attitude toward a website when it is useful for achieving their goals. In contrast, when a website does not provide the information they are looking for, older travelers perceive it to be useless and avoid using it [57,58]. Informative websites provide practical and resourceful information, thus enabling older adults to perceive them as useful and leading them to visit the websites often [56], which increases website familiarity [57].

**Hypothesis** **1** **(H1).** *Travel website informativeness is positively related to older travelers’ website familiarity.*

#### 2.2.2. Effectiveness 

Effectiveness encompasses how information is provided to online consumers [59]. Aspects of a website’s effectiveness include its convenience of use, its layout, and the navigational tools it provides to aid users in their search for products and services [60,61]. Effectiveness also describes the degree to which information is accurate, up to date, complete, and relevant to online shoppers [62].

Older travelers often experience cognitive challenges such as disorientation during web browsing [63], and website effectiveness tools such as navigational functions support them in concentrating while browsing [12]. The cognitive abilities used for internet usage include spatial ability, working memory, and information processing abilities [64,65,66], which decline with age. For example, low spatial ability leads older travelers to feel lost and disoriented within a website [67], and they experience difficulties in learning the spatial structure of a new environment [68]. The navigational functions of a website support older adults in finding what they are looking for in easier ways [12]. Lövdén et al. [69] also found that navigational support is more beneficial to older adults than to younger users.

Furthermore, the disorientation often investigated among older adults can be reduced through website effectiveness. Accurate, relevant, and up-to-date information offered by travel websites can aid users in focusing on their main activity (e.g., searching for information or purchasing a product [13]) and can increase their engagement since the ongoing browsing process leads them to be deeply captivated by the search activity [70]. Therefore, this study infers that website effectiveness assists older travelers in concentrating and engaging more deeply [71], which ultimately leads to familiarity [72].

**Hypothesis** **2** **(H2).** *Travel website effectiveness is positively related to older travelers’ website familiarity.*

#### 2.2.3. Entertainment 

A hedonic web environment has increasingly been identified as important for positive online consumer behavior [73]. Entertainment factors play a major role in creating a hedonic web environment [74] and have therefore become one of the important determinants for site evaluation [51,75]. Entertainment reflects the fun, excitement, and imagination of a website [53]. Kim and Li [50] described entertaining websites as innovative and creative, which make the user feel happy, cheerful, and sociable. Entertaining websites include sensory and hedonic elements such as color, music, photos, and/or interactivity [51] that enable users to perceive them as enjoyable [74]. The appearance and entertainment of websites constitute site quality and enable online consumers to browse and purchase products more easily [52]. 

Socioemotional selectivity theory (SST) posits that older adults prefer emotional-oriented to cognitive-oriented tasks [76]. Consistent with SST, older adults were found to have an emotional dependence on remembered information. For example, Carpenter and Yoon [77] found that presenting information in a hedonic and positive emotional environment increased older travelers’ ability to remember and recall information. Fung and Carstensen [78] also demonstrated that older travelers prefer emotionally meaningful appeals rather than knowledge-based appeals. Based on these findings, it can be inferred that entertaining and hedonic websites support older adults in remembering and recalling information, which increases their familiarity with the website [79].

Moreover, older adults are often motivated by enjoyment in the adoption of new technology [21]. A few studies suggest the importance of entertaining aspects of websites for older travelers since higher levels of entertainment lead to higher levels of website engagement [80,81], which in turn increases website familiarity [49,72]. Kim et al. [82] reported that when seniors feel enjoyment during the use of mobile devices, they become emotionally attached to using them for tourism. Therefore, this study posits that when seniors perceive a website as entertaining, they will feel closeness and an emotional attachment to the website.

**Hypothesis** **3** **(H3).** *Travel website entertainment is positively related to older travelers’ website familiarity.*

### 2.3. Revisit Intention

Loyalty such as repurchase or revisit intention is an inevitable aspect of a brand’s success in the hospitality industry, and many studies explore the revisit intention generation process [83,84,85]. The importance of revisit intention is not an exception for the website and e-commerce success [86]. Revisiting websites reflects customer loyalty, which leads to repurchasing from the website [68,87] without switching to another retailer [15,88,89]. Since an increasing number of older travelers participate in online platforms, studies have explored older adults’ revisit intention to online platforms such as social network sites [82] and mobile social network sites [90]. 

Older adults tend to be risk averse and perceive more risk in the adoption of new technology [54]. For example, older adults perceive more online risks such as computer privacy and security issues than younger groups. Loss aversion theory posits that highly risk-averse people are more sensitive to losses than risk takers [91] and explains that risk-averse consumers tend to avoid shopping at a website they are unfamiliar with [92] to avoid risk. This study thus hypothesizes that older adults will revisit the websites with which they are familiar. Lambert-Pandraud et al. [93] found that senior consumers are likely to remain attached to a brand despite declined innovativeness rather than trying a new brand. Moreover, Simonin and Ruth [29] reported that familiarity has a positive effect on consumers’ intimacy with web-based services and on consumers’ intention to continue using a service. 

Older travelers often require additional time and effort to learn to use the internet due to cognitive declines [90]. Therefore, they are expected to revisit websites they are familiar with since using new websites requires additional cognitive processing to learn how to browse websites, leading to the situation in which the costs of learning new websites exceed the benefits. Flavián, Guinalíu, and Gurrea [88] stated that consumers’ cognitive familiarity, such as their skills in managing and controlling a website, makes it convenient for them to browse a website, thereby reducing the likelihood that they will switch to another website. Söderlund [30] also found that a high level of prepurchase familiarity is associated with repurchase intention.

**Hypothesis** **4** **(H4).** *Website familiarity is positively related to older travelers’ revisit intention.*

### 2.4. Cognitive Age

Aging can be defined chronologically by calendar age; however, aging is not a homogeneous process, and individuals of the same chronological age may be of different cognitive ages [63]. Cognitive age is understood as the age individuals perceive themselves to be [78]; it is a reflection of how they act and their interests [94] and is a useful indicator for individual behavior [26].

According to Staats [25], cognitive age and chronological age tend to be equal for people between the ages of 20 and 50, while people over 50 years old tend to have different cognitive and chronological ages. In addition, cognitive age may also be a better means for understanding and segmenting the older adult market [27]. 

Cognitive age is an important and useful indicator in studies of older consumers’ use of ICT such as the internet or computers [26]. Lian and Yen [95] found that older travelers who have a young cognitive age have a greater willingness to accept and use innovative products. Similarly, Eastman and Iyer [27] reported that older travelers who have a young cognitive age tend to have higher rates of internet usage. Moreover, Gwinner and Stephens [96] reported that older travelers who have a young cognitive age tend to be more involved in new adventures.

This study contends that cognitive age can influence the impact of website atmosphere on website familiarity. According to Gwinner and Stephens [96], cognitive age is negatively related to information seeking, indicating that cognitively young older adults tend to explore more product information. Several existing studies [26,97] similarly found that cognitively young older adults tend to have more willingness to seek information in shopping than their cognitively old counterparts who are reluctant to perform cognitively challenging tasks such as comprehending and accepting new information [98]. This study hypothesizes that cognitively young older adults will feel a closeness to the informative websites providing rich and varied information.

**Hypothesis** **5a** **(H5a).** *Cognitive age moderates the relationship between website informativeness and older travelers’ website familiarity.*

Cognitive age is also associated with cognitive capabilities [99]. Stephan et al. [100] argued that cognitive age is associated with memory decline in capabilities for immediate recall; specifically, older adults who have a younger cognitive age tend to have better immediate recall capabilities. Therefore, they are expected to have better capabilities for recalling information during web browsing, which makes them less distracted and deeply engaged in the browsing activity. The impact of accurate and up-to-date information on older adults’ website familiarity will be accentuated when the cognitively young older adults have better capabilities to recall immediate information.

**Hypothesis** **5b** **(H5b).** *Cognitive age moderates the relationship between website effectiveness and older travelers’ website familiarity.*

Cognitive age may also influence the impact of entertainment aspects of websites on website familiarity. According to Wilkes [101], cognitive age was negatively related to fashion interests and entertainment activity in older women over the age of 60, suggesting that cognitively young older adults are more likely to participate in entertainment-related activities. Moreover, cognitively young older travelers have more willingness to try new and innovative experiences [82]. Given that the entertainment aspects of website atmosphere include creative and innovative ways of displaying and conveying information [50], we hypothesize that older travelers who have a younger cognitive age will feel more comfortable with entertaining and innovative websites.

**Hypothesis** **5c** **(H5c).** *Cognitive age moderates the relationship between website entertainment and older travelers’ website familiarity.*

## 3. Method

### 3.1. Measurement Items

To test the study model empirically, validated and reliable measurement instruments were used from previous studies. Travel website atmosphere contains three subconstructs that were measured by a total of 13 items drawn from the work by Gao and Bai [22]: four for informativeness, four for effectiveness, and five for entertainment. To assess familiarity, four items were adopted from Lee and Kwon [28]. Revisit intention was measured using four items adapted from studies by Chen et al. [15] and Kuo, Wu, and Deng [86]. For the measures of informativeness, effectiveness, entertainment, familiarity, and revisit intention, respondents were asked to score the items from 1 (strongly disagree) to 5 (strongly agree) based on a five-point Likert scale. Additionally, cognitive age was measured by four items derived from a study by Szmigin and Carrigan [26], which asked “I FEEL as though I am in my …,” “I LOOK as though I am in my …,” I DO most things as though I were in my …,” and “My INTERESTS are mostly those of a person in his/her …” (Cronbach’s α = 0.807). Each item assessing cognitive age was anchored using a five-point Likert scale (1 = a lot younger than my age, 5 = a lot older than my age). The questionnaire was completed by revising wordings to fit the context of the travel website through feedback from experts and deleting some items based on the results of the pretest. Cronbach’s α values of all constructs were sufficient, indicating that all measures and constructs were reliable.

### 3.2. Data Collection and Sample

The data for the study came from a survey targeting travelers 50 years of age and older in the United States. The subjects were relatively older e-commerce users, compared with those in previous studies [102,103,104], and participants were included if they were aged 50 or older and had purchased a travel package/activity using a travel website. An online research company, Survey Monkey, distributed 1000 questionnaires using the traveler panel database, and only participants who passed our two screening questions could respond to survey questions (i.e., “Yes” for the first screening question, “Are you over 50?”, and “Yes, and I have purchased a travel package/activity using a travel website” for the second screening question, “Do you use the internet to do research on travel?”). After screening out the unqualified respondents, the qualified participants responded to all questions, receiving a monetary incentive of USD 9.50. A total of 305 responses were valid after deleting extreme responses. Since confirmatory factor analysis requires a minimum sample size of 200 [105], a total of 305 responses in this study were valid to be adopted The mean age of the sample was 59.8 years old, with a range from 50 to 80. The sample profile is provided in Table 1. 

## 4. Results

### 4.1. Measurement Model 

Prior to investigating the structural model, confirmatory factor analysis was performed to assess the validity and reliability of the measures and constructs used in the study model. The measurement model fit for the data was acceptable (χ²= 411.006, *df* = 172, χ²/*df* = 2.296 at *p* < 0.001, normed fit index (NFI) = 0.933, relative fit index (RFI) = 0.921, Tucker–Lewis index (TLI) = 0.954, comparative fit index (CFI) = 0.961, root mean square error of approximation (RMSEA) = 0.065) [106]. 

Convergent validity was assessed by standardized factor loadings for each indicator on its construct and value average variance extracted (AVE) for each latent variable [107]. As shown in Table 2, all standardized loadings were significant and greater than 0.705 (minimum value = 0.5), and all AVE values exceeded the cutoff of 0.5 (see Table 3). Therefore, the convergent validity of the measurements was supported. Discriminant validity indicates that one latent variable is distinctive from the others. According to the suggestion made by Fornell and Larcker [108], a discriminant validity test was performed by comparing the squared correlation estimate between ten pairs of five constructs with the AVE of each construct. Every squared correlation estimate for each pair of constructs was lower than the minimum AVE value for that pair, resulting in satisfactory discriminant validity (see Table 3). Composite reliability values of all latent variables were greater than the recommended criterion of 0.70, supporting the reliability of all measurement items [107].

### 4.2. Structural Model

The structural equation model (SEM) analysis was undertaken to investigate causal relationships between the latent constructs. The results of the analyses revealed that the structural model substantially fit our sample data (χ²= 482.702, *df* = 182, χ²/*df* = 2.652 at *p* < 0.001, NFI = 0.921, RFI = 0.909, TLI = 0.941, CFI = 0.949, RMSEA = 0.074 [106]. 

The results of the SEM analysis with standardized coefficients are presented in Table 4. All three dimensions of travel website atmosphere induced older travelers’ website familiarity: informativeness (β = 0.374, *t*-value = 5.796, *p* < 0.05), effectiveness (β = 0.450, *t*-value = 6.921, *p* < 0.05), and entertainment (β = 0.114, *t*-value = 2.409, *p* < 0.05). Thus, hypothesis 1, hypothesis 2, and hypothesis 3 were supported, and 68.5% of the variance in website familiarity was explained by the three dimensions of website atmosphere. Additionally, older travelers’ familiarity toward specific websites strongly predicted their revisit intention (β = 0.808, *t*-value = 14.081, *p* < 0.05), and website familiarity explained 65.3% of the variance in older travelers’ revisit intention. Thus, hypothesis 4 was supported.

### 4.3. Moderating Effect Test

To test the between-group differences by cognitive age instead of chronological age, a multigroup analysis using SEM was conducted. According to the median value of the sum of the cognitive age ratings, the sample was categorized into a high-cognitive age group (*n* = 140) and low-cognitive age group (*n* = 165). Prior to the SEM test between sub-groups, the measurement equivalence across the groups was assessed [109]. The free model (χ^2^ = 768.413, *df* = 364, χ^2^/df = 2.111, *p* < 0.001, IFI = 0.933, CFI = 0.933, TLI = 0.922, and RMSEA = 0.061) and the constraint model (χ^2^ = 788.581, *df* = 380, χ^2^/df = 2.075, *p* < 0.001, IFI = 0.933, CFI = 0.932, TLI = 0.925, and RMSEA = 0.060) indicated acceptable fit indices. The results revealed that the chi-square difference test was not statistically significant (Δχ^2^ = 20.168 > 26.296 with *df* = 16, *p* < 0.05), indicating full metric invariance across the two groups. 

Subsequently, it was determined if the path coefficients were different between the subgroups by imposing equality constraints on the path coefficients [106]. Table 5 illustrates the results of the moderating function of travelers’ cognitive age on the effects of the three subdimensions of travel website atmosphere on website familiarity. In the relationship between informativeness and website familiarity, the chi-square difference test showed that the free model and constraint model imposed on the path coefficients were significantly different at the 0.05 level (Δχ^2^ = 13.378 > χ^2^ _0.05_(1) = 3.84, *df* = 1). Specifically, in the group with low-cognitive age, the effect of informativeness on website familiarity was stronger than in the group with high-cognitive age (low: β = 0.515, *t*-value = 5.840, *p* < 0.05 vs. high: β = 0.150, *t*-value = 1.570, *p* = 0.116). Thus, hypothesis 5a was supported. However, there were no significant differences at the 0.05 level between the free model and constraint model in the relationships between effectiveness and website familiarity (Δχ^2^ = 2.007 < χ^2^ _0.05_1) = 3.84, *df* = 1), as well as entertainment and website familiarity (Δχ^2^ = 0.015 < χ^2^ _0.05_ (1) = 3.84, *df* = 1). Therefore, hypothesis 5b and hypothesis 5c were not supported. 

**Figure 1:**
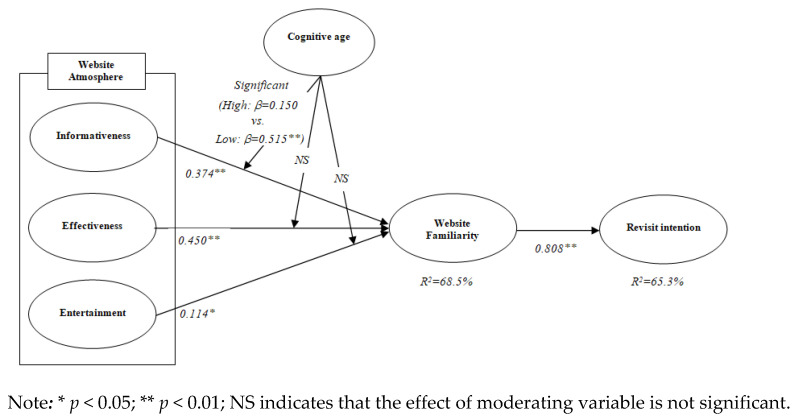
Hypotheses test results.

The analysis results of all hypotheses were illustrated in Figure 1.

## 5. Conclusions

This study investigated the impact of website atmosphere on the older travelers’ website familiarity and revisit intention. This study specifically explored the impact of each website atmosphere element (i.e., informativeness, effectiveness, and entertainment) on older adults’ website familiarity, as well as the moderating effect of cognitive age of each relationship. The main findings of this study as follows. 

First, this study found that informativeness has positive impacts on older travelers’ website familiarity. The result indicates that older travelers tend to feel familiar with the website that provides rich and abundant information on travel products, routes, and/or destinations. One of the major challenges for older travelers in using travel websites is uncertainty regarding the products and/or the websites [57] and a lack of detailed information. An informative website provides extensive information on travel products and travel websites [13], and better knowledge of a website reduces users’ uncertainty regarding the website [39], and thus, travel websites’ informativeness increases older travelers’ familiarity. This finding strengthens the findings of previous studies that found informativeness was one of the major predictors of positive website attitudes [52,53]. 

Second, this study demonstrated that effectiveness has a significant positive impact on older travelers’ website familiarity and was the strongest predictor of older travelers’ website familiarity. Older travelers tend to be reluctant to perform additional cognitive tasks needed to understand complex information [76]. The effective website then reduces the additional time and effort needed for older travelers to understand information, allowing them to feel a familiarity with the website. Moreover, during the ongoing process of information browsing, the effective information leads the users to focus on the website and be deeply captivated by the search activity [70], which results in website familiarity and a positive experience. When the information is inaccurate, irrelevant, or out of date, it may undermine a user’s experience and foster a negative attitude toward the website because they are required to expend more time and effort to accomplish their goal [81], which makes older travelers regard the travel website as an unfamiliar environment. Moreover, the finding that effectiveness has a significant positive impact on older travelers’ website familiarity strengthens the existing argument that consumers who visit travel websites are generally task oriented (i.e., they are concerned about website efficiency and the accuracy of the information provided by the website) [13].

Third, this study demonstrated that entertainment plays a positive role in creating older travelers’ website familiarity and suggests that older travelers tend to feel familiar with a website that uses a variety of sensory and hedonic factors such as color, music, photos, and/or interactivity [51]. This result also supports the findings from existing studies [51,110,111] that found a positive relationship between website design quality and hedonic features of positive online shopping behaviors, such as high levels of shopping enjoyment, shopping concentration [60], actual purchases [12], and consumers’ favorable attitude toward the website [112]. Expanding on the previous finding, the present result shows that entertainment factors create positive travel website familiarity for older travelers. Furthermore, the positive impact of entertainment on website familiarity for older travelers can be explained by a loss in cognitive capability among older adults [27,113], which may result in them relying on images, video, and/or multisensory factors for acceptance of information [77]. 

Fourth, this study confirmed that travel website familiarity strongly predicted revisit intention. Older travelers tend to have revisit intentions to websites with which they are familiar. This study strengthens the findings from existing research that travelers tend to revisit a previous brand by perceiving positive emotions such as hedonic value, mental well-being, or familiarity [114,115,116]. In particular, Lambert-Pandraud, Laurent, and Lapersonne [93] reported that positive emotions such as familiarity elicited by close relationships with a supplier and a biased memory of positive features of a previously chosen option may affect older travelers’ repurchase intention. This study found that older travelers’ preferences for previously purchased brands or shops are not limited to traditional in-store shopping but are also applicable to online shopping. Moreover, several previous studies identified the importance of website familiarity for increasing trust [34] and enhancing loyalty [23], and the current result indicates that older travelers’ website familiarity also influences revisit intention. 

Fifth, more importantly, this study found that the impact of website informativeness is stronger for older travelers who perceive themselves as having a younger cognitive age. Since older travelers who have a younger cognitive age tend to explore, learn, and adopt new information and knowledge through the internet [26,97,98], they respond favorably to and perceive familiarity with websites providing abundant travel information. In contrast, older travelers who have an older cognitive age tend to have more fears in adopting new information [26]; hence, the impact of informativeness on their website familiarity was weaker. 

### 5.1. Theoretical Implications

This study contributes to the online travel literature by investigating older travelers’ perspectives on travel website atmosphere and the moderating role of cognitive age, and it has three specific theoretical implications. 

First, although there have been several studies investigating travel website atmospheres, there has been a paucity of research investigating older adults’ perspectives despite the increasing number of older adults who are tourists and use online travel platforms [18]. This study explored which website atmospheric factors impact older travelers’ familiarity and addressed the suggestions of Wagner, Hassanein, and Head [67], arguing the need for research to consider website design for older travelers’ online shopping. By furthering the knowledge concerning website atmosphere and its influence on website familiarity and revisit intention, this study illuminates the significant moderating role of cognitive age on tourism website usage. 

Second, the results support the literature that highlights the value of cognitive age on older traveler research [27]. The results corroborated the moderating role of cognitive age on the relationship between informative websites and website familiarity and the relationship between website familiarity and revisit intention. The present study expands our understanding of the potential factors for older travelers’ website familiarity since older travelers have stronger familiarity with informative websites when they perceive themselves as cognitively younger. 

Third, this study advances a theoretical understanding of older travelers’ online behavior in the context of a travel website. Although the importance of revisit intention as a significant indicator for e-loyalty has been asserted [15], the previous studies on older travelers’ online behavior mainly focused on the psychological and/or physical challenges of internet usage [57,113,117]. The results of this study found a significant influence of travel website atmosphere on website familiarity, which leads to revisit intention, thereby expanding the current understanding of older travelers’ online usage-related behavior. 

### 5.2. Practical Implications

The present study provides important practical implications for designing travel websites. First, older travelers may prefer websites that provide abundant, valuable, accurate, and up-to-date information. Online travel agencies may need to provide extensive information on destinations, accommodations, and/or airline facilities to attract older travelers. Travel website managers can also use a slider to display photos of attractions as pictures speak louder than words. 

Second, travel websites may adopt an array of browsing and/or search tools that enable older travelers to use online information and perceive ease of use in obtaining information from the internet. Moreover, to maximize older travelers’ familiarity, online travel managers need to obtain relevant information regarding older travelers’ preferences on specific products and/or services as older travelers have website familiarity when the website provides information that matches their goal [56].

Third, travel websites may include various hedonic and sensory functions to create older travelers’ familiarity. Llach et al. [118] similarly recommended that the airline ticket purchasing process includes hedonic aspects such as providing opportunities for fun and enjoyment with website functionalities. For older travelers, a fun website increases their involvement with website browsing activity, which creates website familiarity.

Another implication of this study is the finding that cognitive age has a significant effect on website familiarity and website revisit intention, which provides valuable insight into how marketing strategies of online travel agencies may benefit by recognizing the role of cognitive age in creating website familiarity and facilitating website revisit intention. Targeting older travelers in consideration of their cognitive age rather than their chronological age will be more effective for online travel websites. For example, cognitively younger older adults’ needs and their willingness to try new experiences [27] can be used to develop suitable marketing strategies. 

### 5.3. Limitations and Directions for Future Research

Despite providing an invaluable understanding of travel website atmosphere and older travelers’ perceptions and behavior, this study has several limitations and caution should be exercised before applying the results. First, this study did not assess older travelers’ acceptance and/or dependency level for ICT use. Thus, building on previous work regarding older travelers’ experiences with technology, further study is needed to understand the extent to which older travelers’ website familiarity is influenced by external factors such as website atmosphere and internal factors such as the experience of ICT usage. Moreover, this study employed the concept of cognitive age based on how older travelers identified their age [97]. Future studies can compare the effect of cognitive age with that of chronological age more specifically. 

## Figures and Tables

**Table 1:** Profile of respondents (*n* = 305)**.**

Categories	Variables	*n*	%
Gender	Male	137	44.9
	Female	168	55.1
Age (Mean = 59.8)	50–59	166	54.4
	60–69	107	35.1
	70 and over	32	10.5
Income	Under USD 20,000	31	10.2
	USD 20,000–less than USD 40,000	71	23.3
	USD 40,000–less than USD 60,000	70	23.0
	USD 60,000–less than USD 80,000	82	26.9
	USD 80,000–less than USD 100,000	37	12.1
	Over USD 100,000	14	4.5
Ethnicity	Caucasian	258	84.6
	Hispanic/Latino	10	3.3
	African American	24	7.9
	Asian American	7	2.3
	Other	6	1.9
Education level	Some high school	4	1.3
	High-school graduate	95	31.2
	College or university graduate	151	49.5
	Postgraduate	55	18.0
Marital status	Single	40	13.1
	Married	180	59.0
	Divorced	62	20.3
	Widowed	23	7.6
Current employment	Employed	132	43.3
	Retired	99	32.4
	Semiretired	17	5.6
	Homemaker	17	5.6
	Unemployed	40	13.1

**Table 2:** Confirmatory factor analysis: items and loadings.

Constructs	Items	Cronbach’s α	SL
Informativeness	It is informative to me.	0.937	0.875
	It is a good resource for me.		0.912
	It is useful to me.		0.910
	It provides good knowledge for me.		0.865
Effectiveness	Its information is accurate.	0.919	0.879
	Its information is up to date.		0.891
	Its information is complete.		0.865
	Its information is relevant.		0.808
Entertainment	It is fun to browse.	0.916	0.808
	It is exciting.		0.866
	It is imaginative.		0.874
	It is entertaining.		0.890
	It is flashy.		0.705
Website familiarity	I feel familiar with purchasing goods in the travel website.	0.910	0.776
	I feel familiar with the travel website environment.		0.870
	I feel familiar with the terms used in the travel website.		0.859
	Overall, I feel familiar with the travel website.		0.890
Revisit intention	If I buy tourism products and services again, I would likely buy them from the travel website.	0.934	0.876
	I am likely to return to the travel website for my next purchase.		0.919
	I am likely to make another purchase from the travel website within the next year.		0.841
	I intend to continue using the travel website rather than discontinue its use.		0.898

Note: All factor loadings were significant at *p* < 0.001; SL = standardized loadings.

**Table 3:** Descriptive statistics and correlation matrix.

Variables		*Mean*	*SD*	AVE	CR	1	2	3	4
1	Informativeness	4.065	0.793	0.793	0.969				
2	Effectiveness	3.805	0.742	0.742	0.950	0.712 (0.507)			
3	Entertainment	3.442	0.691	0.691	0.931	0.504 (0.254)	0.467 (0.218)		
4	Website familiarity	3.732	0.722	0.722	0.950	0.718 (0.516)	0.732 (0.536)	0.491 (0.241)	
5	Revisit intention	3.755	0.781	0.781	0.950	0.742 (0.551)	0.780 (0.241)	0.489 (0.239)	0.763 (0.582)

**Table 4:** Standardized parameter estimates for the structural model.

Hypothesis		Paths		Standardized Estimate	*t*-Value	Support
Hypothesis 1	Informativeness	→	Website familiarity	0.374	5.796	Yes
Hypothesis 2	Effectiveness	→	Website familiarity	0.450	6.921	Yes
Hypothesis 3	Entertainment	→	Website familiarity	0.114	2.409	Yes
Hypothesis 4	Website familiarity	→	Revisit intention	0.808	14.081	Yes

**Table 5:** Moderating effect test.

Hypothesis	Paths	High-Cognitive Age (*n* = 140)		Low-Cognitive Age (*n* = 165)		Baseline Model	Restricted Model
		Standardized Estimate	*t*-Value	Standardized Estimate	*t*-Value		
Hypothesis 5a	Informativeness →Website familiarity	0.150	1.570	0.515	5.840 **	χ^2^ (98) = 768.413	χ^2^ (97) = 781.791
Hypothesis 5b	Effectiveness →Website familiarity	0.613	5.501 **	0.313	3.780 **	χ^2^ (98) = 768.413	χ^2^ (97) = 770.420
Hypothesis 5c	Entertainment →Website familiarity	0.148	2.213 *	0.117	1.737	χ^2^ (98) = 768.413	χ^2^ (97) = 768.428
Chi-square difference test: Hypothesis 5a. △χ^2^(1) = 13.378, *p <* 0.05 (significant; hypothesis 5a: supported) Hypothesis 5b. △χ^2^(1) = 2.007, *p >* 0.05 (insignificant; hypothesis 5b: not supported) Hypothesis 5c. △χ^2^(1) = 0.015, *p >* 0.05 (insignificant; hypothesis 5c: not supported)							

Note: * *p* < 0.05; ** *p* < 0.01.

## Data Availability

Data are available on request.
